# pH-Dependent Changes in the Structural and Functional Properties of Quinoa Protein Isolates in Aqueous Dispersions

**DOI:** 10.3390/foods15193501

**Published:** 2026-09-30

**Authors:** Liz Astorga-Oquendo, Federico M. Harte, Osvaldo H. Campanella

**Affiliations:** 1 Department of Food Science, Pennsylvania State University, University Park, PA 16802, USA; astorgaoquendo.1@buckeyemail.osu.edu; 2 Department of Food Science and Technology, The Ohio State University, 2015 Fyffe Road, Columbus, OH 43210, USA

**Keywords:** quinoa protein isolate, pH adjustment, plant-based ingredient, particle size, protein functionality

## Abstract

Quinoa protein isolate (QPI) is an emerging plant-based ingredient with high nutritional quality and significant potential for sustainable and clean-label food formulations. However, limited understanding of the relationships among its major protein fractions, physicochemical characteristics, and functionality restricts its broader utilization in food systems. This study investigated the effects of pH conditions (3, 5, 7, 9, and 11) on the particle size distribution, soluble-solid recovery, interfacial properties (surface hydrophobicity, emulsification, and foaming), and albumin–globulin protein fraction profiles of three QPI aqueous dispersions (Q1, Q2, and Q3). Alkaline conditions significantly enhanced QPI soluble-solid recovery (from <20% to >70%) and interfacial functionality, which were associated with the dissociation of large protein aggregates (D_50_ > 3000 nm) into smaller, well-dispersed particles (D_50_ < 109 nm) and an increased abundance of soluble albumin–globulin protein fractions, particularly chenopodin. SDS-PAGE, densitometry, principal component analysis, and Pearson correlation supported strong relationships among soluble-solid recovery, particle size reduction, interfacial properties, and soluble albumin–globulin protein fractions. These findings provide an integrated understanding of pH-dependent structure–function relationships of quinoa proteins and demonstrate the potential of pH adjustment for modulating the functionality of QPI aqueous dispersions for sustainable, clean-label ingredients in emulsified foods, aerated systems, and next-generation protein-rich formulations.

## 1. Introduction

Consumers increasingly demand processed foods with clean labels, i.e., foods that contain minimal and familiar ingredients that are free from synthetic additives [1,2]. Meeting this demand is challenging for plant-protein-based foods because soy proteins remain the primary plant protein ingredient used in the food industry, while the functionality of many alternative plant protein sources remains insufficiently characterized [3]. In parallel, consumer interest in novel protein sources continues to grow. For example, a Mintel survey found that, among U.S. consumers of plant-based meat substitutes, 44% preferred quinoa as a protein source compared with 34% who preferred soy [4]. This trend reflects the increasing recognition of quinoa as a high-quality plant protein with a well-balanced essential amino acid profile [4,5]. Consequently, diversifying plant protein sources through the incorporation of novel crops such as quinoa is increasingly viewed as a strategy to improve the sustainability, nutritional quality, and resilience of the global food supply. Owing to its strong potential, the Food and Agriculture Organization of the United Nations identified quinoa as one of the crops that could contribute to food security in the 21st century [5,6,7].

Quinoa (*Chenopodium quinoa*), a pseudocereal native to South America, has significant economic potential in the food industry due to its high-quality protein composition and favorable nutritional profile. Its proteins contain essential amino acids, including cysteine, lysine, and tryptophan, which are often limited to other plant sources. Additionally, quinoa is rich in vitamins and minerals, offers a gluten-free alternative, and is considered hypoallergenic. Its low prolamin content makes it particularly suitable for individuals with celiac disease or gluten intolerance [7,8,9,10]. Beyond its nutritional quality, quinoa protein has also gained attention for its potential biological activity, as peptides generated during protein digestion have been reported to exhibit antioxidant activity [11].

Quinoa flour is the primary raw material used for the production of quinoa protein isolate (QPI). According to the Osborne classification, quinoa proteins are primarily composed of 2S albumins (35%) and 11S globulins (37%), with only minor amounts (0.5–7%) of prolamins [7]. Chenopodin, the major 11S globulin fraction in quinoa, is a hexameric protein with a molecular size in the range of 300–360 kDa, consisting of basic and acidic polypeptides linked by disulfide bonds. The 2S albumin fraction comprises a mixture of low molecular weight polypeptides in the range of 8–9 kDa [12,13]. More recently, 7S globulins have been identified in quinoa proteins, consisting of peptide sequences ranging from 16–66 kDa that assemble as trimers or tetramers [14].

Quinoa proteins have received considerable attention from food processors because they contribute desirable textural and structural attributes, in addition to flavor and color, to formulated foods [6]. However, their physicochemical properties depend primarily on protein structure, which can be altered by environmental conditions, particularly pH [15,16]. Changes in pH modify protein charge and can influence key functional properties, including solubility, emulsifying and foaming properties, and water-binding capacity, which ultimately dictate their potential food applications [7,13].

Several studies have investigated the effects of extraction pH or pH-shifting treatments on the physicochemical and functional properties of quinoa proteins. More recently, Cui et al. [17] demonstrated that extraction pH (7–11) affected QPI protein recovery, solubility, interfacial properties, and heat-induced aggregation behavior. Similarly, Wang et al. [18] reported that pH-shifting alone reduced QPI particle size from approximately 2700 nm at pH 7 to 1330 and 755 nm following treatment at pH 10 and 12, respectively, while also substantially increasing protein solubility, particularly following alkaline pH-shifting. Solanilla-Duque et al. [19] further demonstrated pH-dependent differences in the interfacial, rheological, emulsifying, and foaming behavior of QPI at pH 5 and 7. Elsohaimy et al. [16] reported that QPI solubility was highly pH-dependent, reaching a maximum value of 75.2% at pH 10 and a minimum value of 25.6% at pH 4. Similarly, Foste el al. [20] observed the lowest solubility (<65%) under acidic conditions (pH 2–4). Abugoch et al. [21] reported that QPI solubility exceeded 90% at pH 11 and was substantially lower at pH 3; however, water-holding capacity (WHC) remained relatively constant between pH 3 and 7, suggesting that solubility and WHC are not directly related. Shen et al. [22] found that the foaming capacity was higher (>100%) at pH 8 and lower (approximately 60%) at pH 5. Likewise, Ruiz et al. [12] reported greater solubility of QPI at pH 9 (>50%) and lower solubility at pH 5. Despite these findings, comparatively less information is available on the pH-dependent behavior of different QPI materials evaluated under identical aqueous conditions, particularly regarding the relationships among particle size distribution, soluble albumin–globulin protein fractions (including chenopodin), soluble-solid recovery, and interfacial functionality. Such knowledge is critical for optimizing the formulation, stability, and performance of quinoa-based food systems.

Therefore, the objective of this study was to comparatively evaluate three QPIs from different commercial sources under identical aqueous pH conditions (pH 3, 5, 7, 9, and 11) to determine their pH-dependent particle size characteristics, soluble-solid recovery, surface hydrophobicity, interfacial properties, and soluble albumin–globulin protein fraction profiles. Multivariate analyses were further used to examine relationships among these physicochemical and functional responses. By integrating these measurements across different QPI materials under the same pH conditions, this study provides a comparative assessment of their pH-dependent physicochemical and functional behavior and contributes to understanding the relationships among dispersion characteristics, soluble protein fractions, and interfacial functionality relevant to quinoa-based food applications.

## 2. Materials and Methods

### 2.1. Materials

Quinoa flour was purchased from three commercial suppliers: Kapok Naturals (Laboratorios Portugal, Arequipa, Peru), Livekuna (Kunachia LLC, Quito, Ecuador), and Andina Organic (Bolicert, La Paz, Bolivia). Based on the nutritional information reported on the product labels, the protein contents of the quinoa flours from Peru, Ecuador, and Bolivia were 15.2, 14.3, and 13.0 g/100 g, respectively. The corresponding fat contents were 6.1, 7.1, and 5.0 g/100 g, and the carbohydrate contents were 63.6, 64.3, and 70.0 g/100 g, respectively. These compositional values were not independently determined in this study. All other chemicals were of analytical reagent grade.

### 2.2. Isolation of Quinoa Protein from Quinoa Flour

Quinoa protein isolates (QPIs) were obtained from quinoa flour samples following the method described by Abugoch et al. [21] with slight modifications. Briefly, quinoa flour was defatted three times with hexane (Merck, KGaA, Darmstadt, Germany) at a flour-to-solvent ratio of 1:4 (*w*/*v*) with continuous stirring overnight at 22 ± 2 °C. To remove residual hexane, the defatted flour slurry was placed in a fume hood for 2 days.

Defatted flour dispersions were prepared by suspending the flour in DI water at 10% (*w*/*v*) and mixing thoroughly. The pH of the dispersion was adjusted to 10 using 1 M NaOH. The suspensions were stirred for 1 h at 23 °C, followed by centrifugation at 10,000× *g* for 20 min at 20 °C using an S50-A rotor (Sorvall MTX 150, Thermo Scientific, Asheville, NC, USA). After removal of the precipitate, the supernatant was collected, and the pH was adjusted to 4.5 with 2 M HCl to precipitate the protein. The precipitated protein was recovered by centrifugation at 10,000× *g* for 20 min at 20 °C.

The precipitated protein pellets were resuspended in DI water and neutralized to pH 7 with 0.2 N NaOH. The suspensions were then freeze-dried using a Virtix Genesis Pilot freeze dryer (SP Scientific, Warminster, PA, USA) for 72 h. The obtained freeze-dried quinoa protein isolates (QPIs) were stored at 4 °C until further use and were designated as Q1, Q2, and Q3, representing the protein isolates obtained from the Peruvian, Ecuadorian, and Bolivian quinoa flours, respectively. Some of the experimental data presented in this study were previously included in the first author’s M.S. thesis [23]. The present study expands upon this work through additional data analyses and a more comprehensive evaluation of the relationships among QPI structure, soluble-solid recovery, and interfacial functionality under different pH conditions.

### 2.3. Proximate Composition of QPIs

The nitrogen content of QPIs was determined using the Dumas combustion method [24] and a LECO FP-528 nitrogen analyzer (LECO Corp., St. Joseph, MI, USA). Briefly, QPI samples were weighed (200 mg), transferred to a thin foil capsule, and loaded into the carousel of the instrument for combustion. Protein content was calculated using a nitrogen conversion factor of 6.25, as previously employed for quinoa protein [12,25].

The moisture content of the freeze-dried QPIs was evaluated using a moisture analyzer (Model MA37, Sartorius Lab Instruments, Gottingen, Germany). A representative sample of freeze-dried QPI was weighed, loaded into an aluminum sample pan, and placed into the analyzer for moisture determination according to the manufacturer’s instructions.

### 2.4. Preparation of QPI Aqueous Dispersions at Different pH Conditions

Freeze-dried QPIs were dispersed in DI water at a concentration of 1% (*w*/*v*). The dispersions were stirred using a magnetic stirrer for 12 h at ambient temperature (21 °C) to ensure protein hydration. Subsequently, the pH of the QPI aqueous dispersions was adjusted to pH 3, 5, 7, 9, and 11 by the addition of 2 M HCl or 2 M NaOH. The pH was monitored using a pH meter (Model Ion 700, Oakton, Vernon Hills, IL, USA) equipped with a probe electrode. Following pH adjustment, the QPI aqueous dispersions were maintained at their respective pH conditions and subjected to the analyses described in the following sections.

### 2.5. Viscosity of QPIs

Rheological measurements of QPI aqueous dispersions subjected to different pH treatments were assessed using a Discovery HR-3 rheometer (TA Instruments, New Castle, DE, USA) equipped with a double-gap concentric cylinder geometry. Flow curves (shear stress vs. shear strain rate) were obtained over a shear rate range of 1–100 s^−1^ at 20 °C. The viscosity values reported in this study were obtained at a shear rate of 100 s^−1^. The resulting flow curves were used to assess the effect of pH on the viscosity behavior of the QPI dispersions.

### 2.6. Particle Size Distribution of QPIs

**Figure 1:**
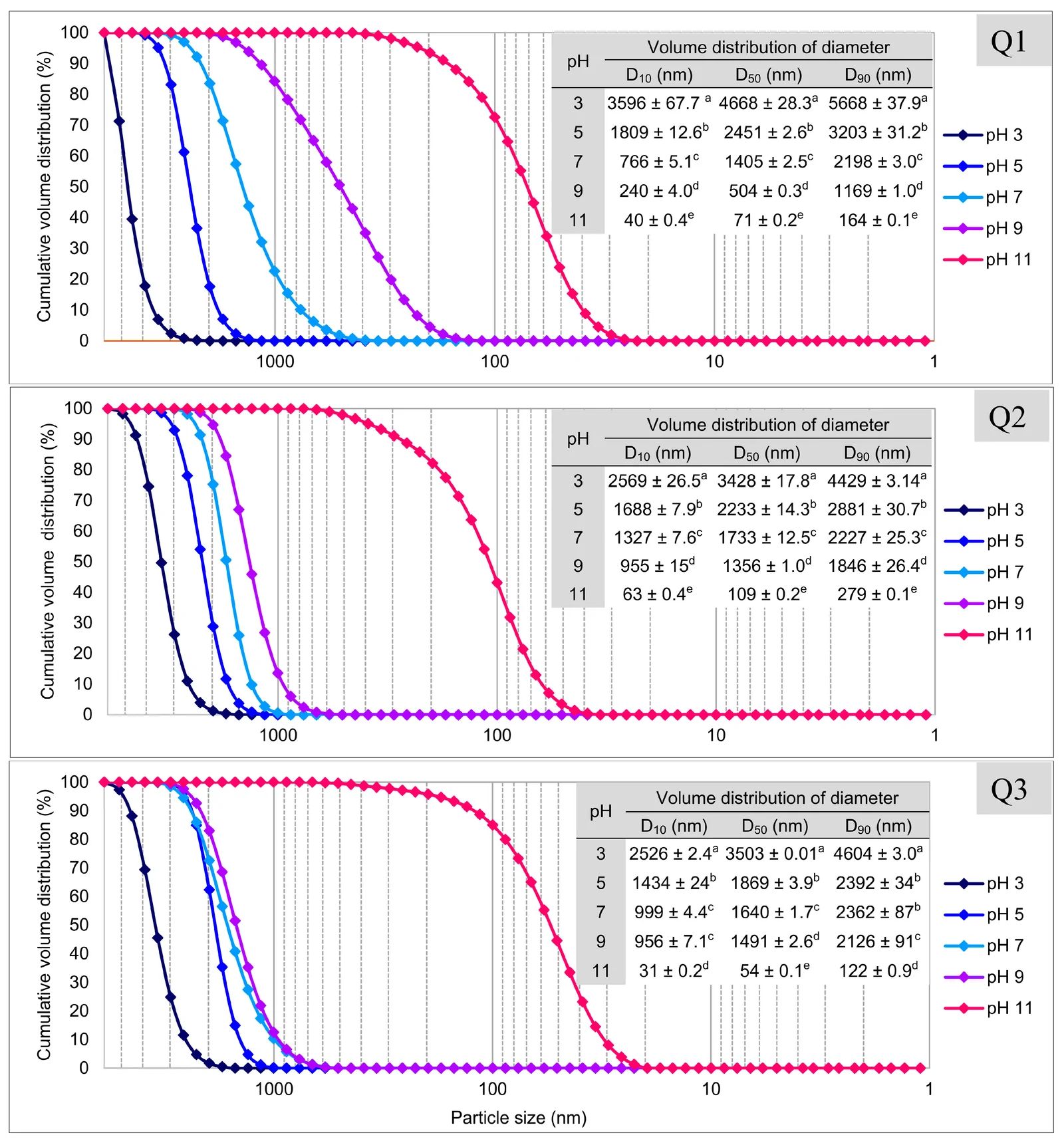
Cumulative volume-based particle size distribution in a logarithmic plot for quinoa protein isolates (Q1, Q2, and Q3) in aqueous dispersions with different pH treatments. The insert shows the volume distribution diameters for D_10_, D_50_, and D_90_. Values are given as mean ± SD (n = 3). Means followed by different superscript letters indicate a significant (*p* < 0.05) difference (Tukey’s HSD test).

The particle size distribution of QPI aqueous dispersions subjected to different pH treatments was determined by dynamic light scattering (DLS) using a particle size analyzer (Horiba LB-550, Irvine, CA, USA) at 22 °C. A refractive index (RI) of 1.45 was used for all analyses. Both the cumulative volume-based particle size distribution and the density-based particle size distribution (q3%) were determined and plotted on a logarithmic scale. The cumulative volume-based distribution is presented in Figure 1, whereas the density-based distribution (q3%) is provided in Supplementary Figure S1. The volume-based particle size percentiles (D_10_, D_50_, and D_90_) were also reported to describe the distribution of particle sizes within the samples.

### 2.7. Surface Hydrophobicity (H_o_) of QPIs

The surface hydrophobicity (H_o_) of the QPI aqueous dispersions subjected to different pH treatments was determined according to the method of Haskard and Li-Chan [25], using 8-anilino-1-naphthalene sulfonic acid (ANS) as a fluorescence probe. An 8 mM ANS stock solution was prepared in phosphate buffer (pH 7.0). For the analysis, 20 μL of ANS stock solution and 4 mL of phosphate buffer were added to successive sample aliquots containing 10, 20, 30, 40, or 50 μL of the QPI dispersions.

The mixtures were vortexed and incubated in the dark at 22 °C for 15 min. Fluorescence intensity (FI) was measured using a Cary Eclipse spectrofluorometer (Agilent Technologies Inc., Mulgrave, Australia) at an excitation wavelength of 388 nm and an emission wavelength of 470 nm. Surface hydrophobicity (H_o_) was estimated from the initial slope of the fluorescence intensity versus protein concentration plot obtained by linear regression analysis.

### 2.8. Soluble-Solid Recovery of QPIs

The soluble-solid recovery of QPIs subjected to different pH treatments was determined according to the method described by Astorga-Oquendo et al. [26]. Briefly, 20 mL aliquots of QPI dispersions were centrifuged at 50,000× *g* for 1 h at 20 °C, after which the supernatants were collected. Known weights of the original dispersions and the corresponding supernatant were dried at 102 °C to constant weight to determine their total solids content.

Soluble-solid recovery of the QPIs was calculated as the percentage ratio of the total solids present in the supernatant to the total solids present in the original dispersion, according to Equation (1):

(1) $$\mathrm{Soluble}-\mathrm{solid}\;\mathrm{recovery}=\left(\frac{\mathrm{Total solids of supernatant}}{\mathrm{Total solids of original sample}}\right)\times 100$$

### 2.9. Turbiscan Stability Index (TSI) of QPIs

The emulsion stability of QPI samples subjected to different pH treatments was evaluated using the Turbiscan Stability Index (TSI), which provides a quantitative measure of the overall destabilization of emulsion over time. Emulsions were prepared by mixing commercial soybean oil with QPI aqueous dispersions at an oil-to-dispersion volume ratio of 1:4 at ambient temperature (21 °C). The mixtures were homogenized using a high-speed homogenizer (Ultra-Turrax, TP 18–10, IKA Works, Staufen, Germany) operating at 20,000 rpm for 1 min. Immediately after homogenization, the resulting emulsions were transferred to a Turbiscan Lab Expert (Formulaction, Toulouse, France), which monitors sample stability by measuring transmitted and backscattered light at a wavelength of 880 nm. The instrument was programmed to scan the entire height of each sample at 20 min intervals over a total of 6 h. Changes in the transmission and backscattering profiles over time were analyzed using TurbiSoft Lab software, version 2.2, to calculate the Turbiscan Stability Index (TSI), a device-specific parameter that indicates emulsion destabilization, defined by Equation (2):

(2) $$\mathrm{TSI}=\sum_{i}\frac{\Sigma_{h}\;|{\mathrm{Scan}}_{i}\;\left(h\right)-{\mathrm{Scan}}_{i-1}\;(h)\;|}{H}$$

where *Scan_i_* (*h*) is the signal recorded during scan *_i_*(*h*) at a given sample height *h*, whereas *Scan_i_*_−1_ (*h*) is the signal recorded during the previous scan at the same height, and *H* is the total height of the sample. Lower TSI values indicate greater overall emulsion physical stability, whereas higher TSI values indicate greater overall physical destabilization. Because initial emulsion droplet-size distribution and ζ-potential were not determined in this study, TSI was used as an overall stability indicator and was not used to distinguish among specific destabilization mechanisms.

### 2.10. Foaming Properties of QPIs

The foaming capacity (FC) of the QPI aqueous dispersions subjected to different pH treatments was determined by transferring a 10 mL sample into a 50 mL tube (VWR International LLC, Radnor, PA, USA) and vigorously vortexing the dispersion for 1 min at 3200 rpm. The foaming capacity of QPI aqueous dispersions was calculated according to Equation (3):

(3) $$\mathrm{FC}=\left(\frac{V_{o}-V_{\mathrm{liquid}}}{V_{\mathrm{liquid}}}\right)\times \;100$$

where *V_o_* is the foam volume (mL) obtained after vortexing, and *V_liquid_* is the initial volume of the sample obtained before vortexing.

Foaming stability was calculated using the foam volume stability index (FVSI). To determine FVSI, 10 mL of each QPI dispersion was vigorously vortexed at 3200 rpm for 1 min to generate foam. The resulting foam was allowed to stand undisturbed at room temperature (21 °C), and the total foam volume, including the drained liquid phase, was recorded every hour for 10 h. The FVSI was calculated according to Equation (4):

(4) $$\mathrm{FVSI}=\left(\frac{V_{t}-V_{\mathrm{liquid}}}{V_{o}-V_{\mathrm{liquid}}}\right)\times 100$$

FVSI is the foam stability over time, *V_t_* is the total foam volume (mL), including the drained liquid phase, at a given time, *V_o_* is the initial foam volume (mL), and V_liquid_ is the initial volume of the QPI dispersion (10 mL).

### 2.11. Sodium Dodecyl Sulfate-Polyacrylamide Gel Electrophoresis (SDS-PAGE) of QPIs

SDS-PAGE analysis of QPI aqueous dispersions was conducted under reducing and denaturing conditions to evaluate protein profiles before and after ultracentrifugation at 50,000× *g* for 1 h at 20 °C. Ultracentrifugation was carried out to separate the soluble protein fraction (supernatant) from aggregated proteins (pellet), enabling characterization of both soluble and aggregated proteins within the dispersion.

SDS-PAGE was performed according to the Bio-Rad protocol (Bio-Rad Laboratories, Hercules, CA, USA). Briefly, 10 μL of the sample was mixed with 10 μL of sample buffer containing 0.5 M Tris-HCl buffer at pH 6.8, 10% (*w*/*v*) sodium dodecyl sulfate (SDS), 2% 2-β-mercaptoethanol, 0.5% (*w*/*v*) bromophenol blue, and glycerol. The mixtures were heated in a boiling water bath for 6 min and cooled before loading 15 μL of sample into the wells of 12% TGX precast gels (Bio-rad Laboratories, CA, USA). Additionally, molecular ladders ranging from 10 to 250 kDa (Bio-rad Laboratories, CA, USA) were loaded into the first well of each gel to be used as reference standards. Electrophoresis was carried out in 1× Tris/glycine–SDS running buffer at a constant voltage of 140 V. Following electrophoresis, the gels were stained with Coomassie Brilliant Blue R-250 for 1 h and subsequently destained overnight. Protein band intensities from the SDS-PAGE gels were quantified using ImageJ software (version 1.52, National Institutes of Health, Bethesda, MD, USA).

### 2.12. Statistical Analysis of the Data

Unless otherwise stated, all measurements were performed using three independent experimental replicates per treatment (n = 3), with each replicate prepared and analyzed separately. Data were analyzed using a completely randomized experimental design. Analysis of variance (ANOVA) was conducted to identify significant treatment effects, and treatment means were compared using Tukey’s Honestly Significant Difference (HSD) test. Statistical analyses were performed using SAS Studio (version 9.4, SAS Institute Inc., Cary, NC, USA). Results are reported as mean ± standard deviation. Pearson correlation analysis and principal component analysis (PCA) were performed to evaluate multivariate relationships among quality parameters. Correlation matrices and PCA biplots were generated using the Seaborn, version 0.13.2, Matplotlib, version 3.10.3, and Scikit-learn libraries within Google Colaboratory (Google LLC, Mountain View, CA, USA).

## 3. Results and Discussion

### 3.1. Composition of Freeze-Dried Quinoa Protein Isolates (QPIs)

Freeze-dried quinoa protein isolates (QPIs) prepared from commercial quinoa flour samples were labeled as Q1, Q2, and Q3. The moisture and protein contents of the freeze-dried isolates are summarized in Table 1.

Moisture content ranged from 2.7% to 6.5%, whereas protein content varied between 85.9% and 96.0%. These values are consistent with those reported in previous studies using similar extraction protocols [27,28]. The observed differences in moisture and protein content among the QPIs may reflect variability in the composition and characteristics of the commercial quinoa flours used as starting materials. Although manufacturer-reported nutritional information was available for the starting flours, their composition was not independently characterized in this study. Factors such as sample origin, cultivation environment, postharvest handling, and grain composition could potentially contribute to variability among the resulting isolates; however, information on these factors was not available, and their relative contributions cannot be determined from the present study.

### 3.2. pH-Dependent Changes in Particle Size Distribution of QPI Aqueous Dispersions

The effect of pH on the particle size distribution of QPI aqueous dispersions is illustrated in Figure 1. Particle size was characterized using the volume-based diameter parameters (D_10_, D_50_, and D_90_), together with the cumulative volume-based particle size distribution. The D_10_, D_50_, and D_90_ parameters correspond to the particle diameters below which 10%, 50%, and 90% of the total particle volume are contained, respectively. The cumulative volume-based particle size distribution describes the proportion of total particle volume across the measured size range.

For all protein isolates, the median particle size diameter (D_50_) decreased linearly with increasing pH. On average, D_50_ decreased by approximately 430 nm for each unit increase in pH over a pH range of 3 to 11 (D_50_ = 4793 − 432 × pH; R = 0.94). However, differences in pH responsiveness were observed among the isolates. For Q1, all particle size parameters (D_10_, D_50_, and D_90_) decreased progressively with increasing pH, indicating a gradual dissociation of protein aggregates across the entire particle size distribution. Conversely, samples Q2 and Q3 exhibited relatively small changes in D_10_, D_50_, and D_90_ between pH 3 and 9, followed by a pronounced reduction at pH 11. This behavior was particularly evident for D_90_, which represents the larger particles within the dispersions. For example, D_90_ decreased from 1846 and 2126 nm at pH 9 to 279 and 122 nm at pH 11 for Q2 and Q3, respectively. Similar trends were observed for D_10_ and D_50_, suggesting that highly alkaline conditions promoted extensive disaggregation across the entire particle size distribution. These results indicate that Q2 and Q3 required stronger alkaline conditions to dissociate protein aggregates, whereas Q1 exhibited a more gradual reduction in aggregate size with increasing pH, highlighting differences among quinoa protein isolates from different origins.

In agreement with previous reports, alkaline conditions promoted the dissociation of QPI aggregates, whereas acidic conditions favored large aggregate formation [6,29]. All QPI dispersions (Q1, Q2, and Q3) exhibited unimodal log-normal particle size distributions (Figure S1), indicating the presence of a dominant particle population under all pH conditions.

The larger protein sizes observed at pH 3 and 5 can be attributed to the proximity of these conditions to the isoelectric point of quinoa proteins. Near the isoelectric point, the net charge on the proteins is close to zero, minimizing electrostatic repulsion between protein molecules and promoting protein–protein interactions. Consequently, these aggregates result in larger protein sizes, leading to increased particle sizes and, in some cases, reduced solubility. Conversely, under alkaline conditions (pH 9 and 11), proteins carried a greater net negative charge due to the ionization of acidic amino acid residues. The resulting increase in electrostatic repulsion reduced intermolecular association and favored the dissociation of protein aggregates, resulting in smaller apparent particle sizes. At neutral pH, proteins possessed an intermediate net charge, resulting in moderate electrostatic repulsion and partial aggregation. Accordingly, particle sizes at pH 7 were intermediate between those observed under acidic and alkaline conditions. These findings demonstrate that pH-dependent changes in protein charge strongly influenced the aggregation behavior and particle size distribution of QPI dispersions.

### 3.3. pH-Dependent Surface Hydrophobicity of QPI Aqueous Dispersions

The fluorescence emission spectra of the hydrophobic fluorescent probe, ANS, were used to determine the surface hydrophobicity (H_0_) of QPI aqueous dispersions. Due to its high sensitivity to the polarity of the surrounding environment, ANS is widely used to detect protein conformational changes, including unfolding and denaturation. Therefore, changes in the exposure of hydrophobic protein regions can be used to assess structural modification affecting protein functionality [30].

**Figure 2:**
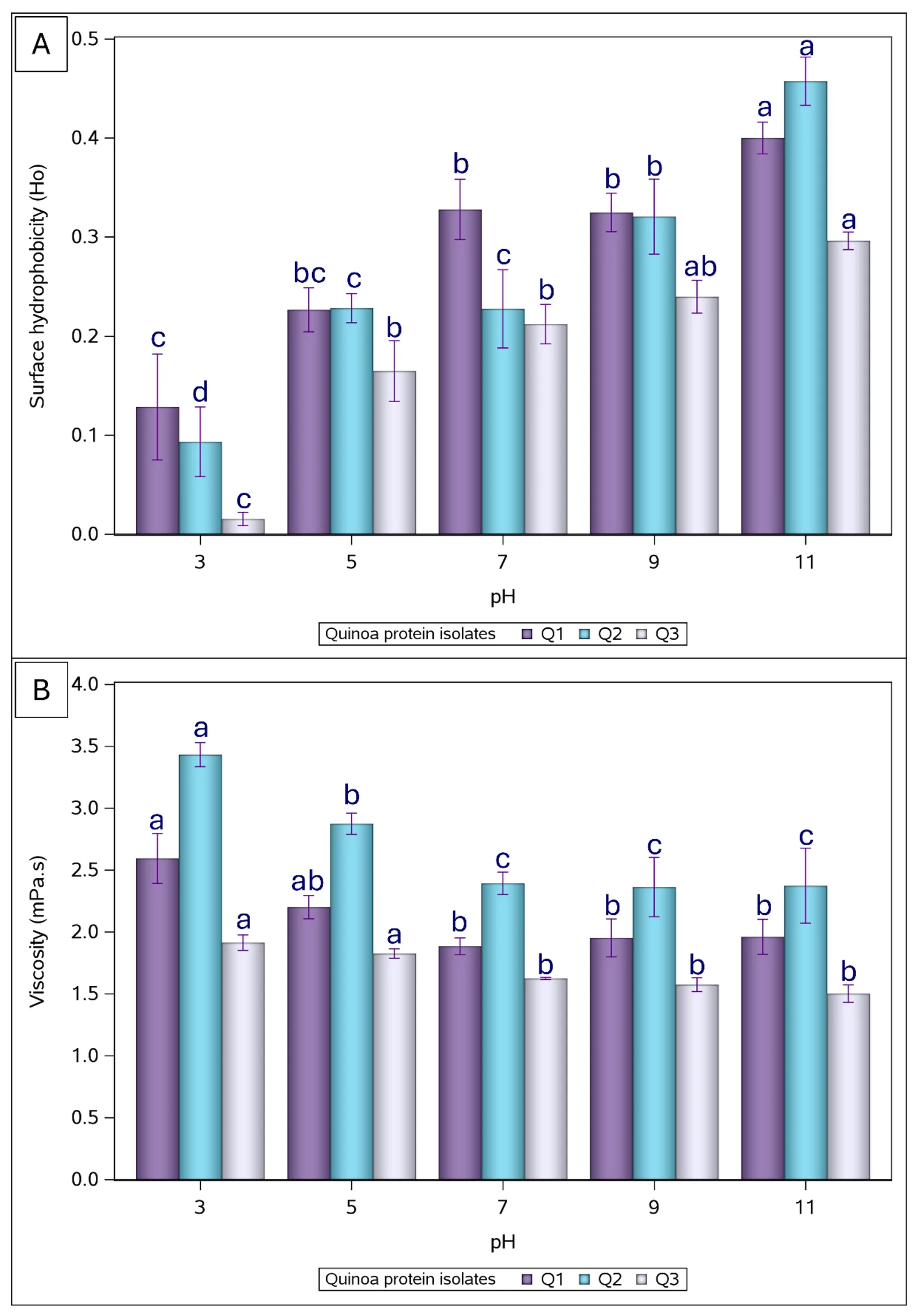
(**A**) Surface hydrophobicity and (**B**) viscosity of QPI aqueous dispersions. Values and error bars indicate mean ± SD (n = 3). Superscript letters indicate a significant (*p* < 0.05) difference (Tukey’s HSD test).

As shown in Figure 2A, the surface hydrophobicity of QPI aqueous dispersions increased significantly (*p* < 0.05) with increasing pH, reaching the highest values at pH 11. Under these strong alkaline conditions, QPI molecules carry a strong net negative charge, leading to pronounced electrostatic repulsion that outweighs hydrophobic interactions. This repulsion can promote partial unfolding of the protein structure, exposing internal hydrophobic regions normally buried within the protein matrix, making them accessible to ANS binding and resulting in increased fluorescence.

Notably, the increase in surface hydrophobicity was accompanied by a reduction in particle size, suggesting that aggregate dissociation and partial protein unfolding may have occurred simultaneously under alkaline conditions. As protein aggregates dissociated into smaller particles, previously buried hydrophobic regions became exposed to the surrounding medium, contributing to the increase in H_0_. These observations are consistent with previous studies [31,32], which reported increased surface hydrophobicity of ginkgo seed protein isolates (GSPI) and soy protein isolates (SPI) subjected to extreme alkaline conditions due to the exposure of hydrophobic groups previously located within the native protein structure.

### 3.4. pH-Dependent Viscosity of QPI Aqueous Dispersions

The viscosity of QPI aqueous dispersions under different pH conditions is illustrated in Figure 2B. All QPI samples (Q1, Q2, and Q3), prepared as 1% (*w*/*v*) aqueous dispersions, exhibited Newtonian flow behavior over the pH range studied (3–11). At pH 7, the viscosities of Q1, Q2, and Q3 were 1.9 ± 0.1 mPa·s, 2.4 ± 0.1 mPa·s, and 1.6 ± 0.0 mPa·s, respectively. Increasing the pH to alkaline conditions did not significantly (*p* > 0.05) affect viscosity compared to viscosities measured at pH 7, despite the substantial reduction in particle size observed in Figure 1.

Conversely, the viscosity of QPIs increased significantly (*p* < 0.05) under acidic conditions. At pH 3, the viscosities of Q1, Q2, and Q3 were 2.6 ± 0.2, 3.4 ± 0.1, and 1.9 ± 0.1 mPa·s, respectively. The higher viscosity at acidic pH is likely associated with the formation of clusters of large protein aggregates resulting from the reduced electrostatic repulsion near the isoelectric point. These larger aggregates are likely to increase the effective hydrodynamic volume of the dispersed phase and promote intermolecular interactions, leading to greater resistance to flow. This explanation is consistent with the particle size results, which showed the highest D_10_, D_50_, and D_90_ values at pH 3 and 5. Interestingly, although alkaline conditions were associated with reduction in particle size, viscosity remained similar to that observed at neutral pH. This suggests that the reduction in particle size was not sufficient to alter the flow behavior of the relatively dilute (1%, *w*/*v*) protein dispersions tested. Therefore, while pH strongly influenced the aggregation state of QPI particles, its effect on viscosity was only evident under acidic conditions where large protein aggregates were observed.

### 3.5. pH-Dependent SDS-PAGE Profiles of QPI Aqueous Dispersions Before and After Ultracentrifugation

**Figure 3:**
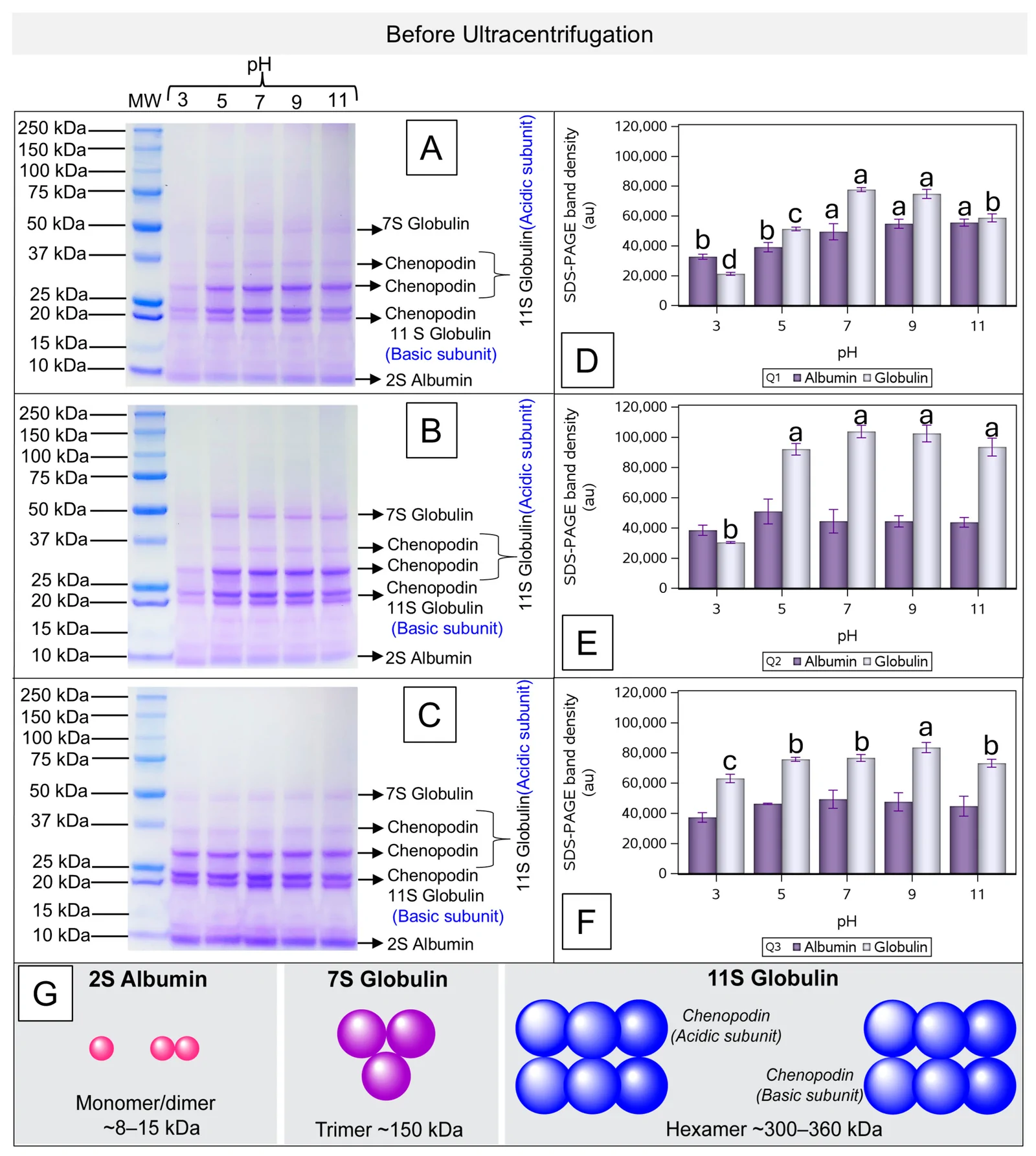
SDS-PAGE of quinoa protein isolate (QPI) dispersions at different pH values for (**A**) Q1, (**B**) Q2, and (**C**) Q3 before ultracentrifugation. Molecular markers (kDa) were used in the first lane. Corresponding densitometry analyses obtained using ImageJ software are shown in (**D**–**F**), with values presented as mean ± SD (n = 3). Superscript letters indicate a significant (*p* < 0.05) difference (Tukey’s HSD test). (**G**) Schematic representation of the main quinoa storage proteins (albumins and globulins).

SDS-PAGE analysis of QPI aqueous dispersions at five pH conditions was performed before ultracentrifugation to characterize the protein polypeptide profiles of the QPI samples. The SDS-PAGE examination of the total protein profile for QPIs is shown in Figure 3A–C. Distinct protein bands corresponding to previously identified quinoa storage proteins were observed. Based on early studies [8,33], these bands were assigned to 2S albumins, 7S globulins, and 11S globulins. The 2S albumins were detected at a molecular weight of approximately 8–9 kDa, while the 7S globulins appeared near 50 kDa. The 11S globulin, commonly referred to as chenopodin in quinoa, is a major storage protein and displays bands corresponding to basic polypeptide subunits (22–23 kDa) and acidic polypeptide subunits (31–37 kDa). These observations agree with a previous report by Brinegar and Goudan [34], which described quinoa 11S globulins as oligomeric proteins composed of heterogeneous acidic and basic subunits linked by disulfide bonds in their native structure (Figure 3G).

Following the identification of the major protein fractions, their relative abundance was estimated by densitometric analysis (Figure 3D–F). Based on band densitometric quantification, the globulin fraction was significantly (*p* < 0.05) more abundant than the albumin fraction in all QPI samples, consistent with previous reports describing globulins as the predominant storage proteins in quinoa seeds [8,13].

It is worth noting that the alkaline extraction method and the freeze-drying procedures used to obtain the protein isolates from flour samples preserved the major storage proteins of quinoa (albumins and globulins). Similar banding patterns and relative abundance of albumin- and globulin-associated polypeptides were observed in Q1, Q2, and Q3 despite their different flour origins, indicating that the extraction process did not substantially alter the distribution of quinoa storage proteins. Overall, the protein profiles obtained in this study were consistent with those previously reported for quinoa protein isolates [8,33].

Although previous studies have used SDS-PAGE to study the effect of pH on quinoa proteins, the composition of the soluble protein fraction following pH-induced aggregation has received limited attention. To address this knowledge gap, QPI aqueous dispersions were ultracentrifuged before SDS-PAGE analysis, thereby allowing separation of the soluble fraction (supernatant) from the aggregate fraction (pellet). The supernatant contained proteins that remained dispersed under each pH condition, while the pellet included aggregated and precipitated proteins. This fractionation approach enabled a more detailed analysis of how pH affects the distribution and solubility of specific quinoa protein subunits.

**Figure 4:**
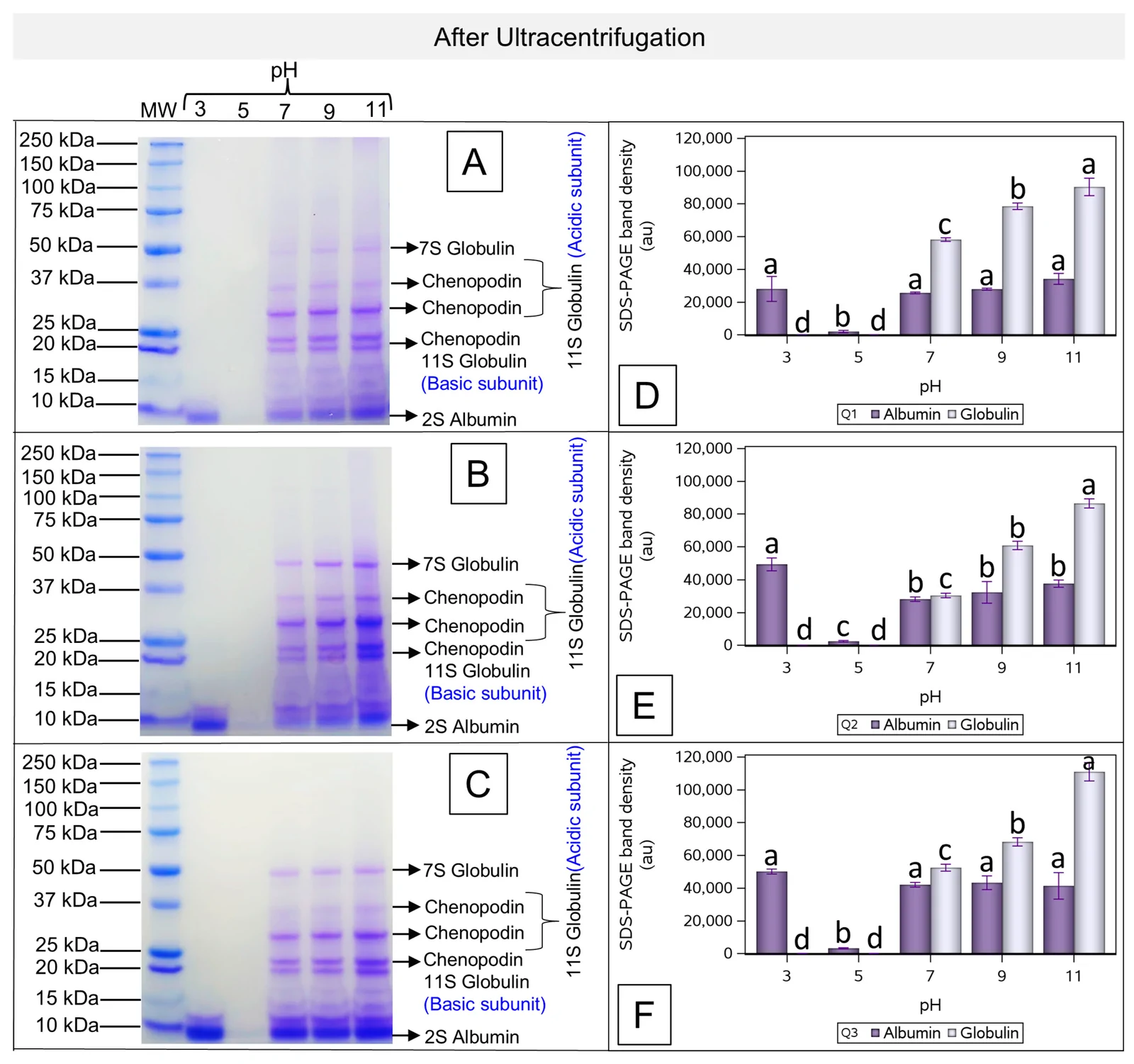
SDS-PAGE of quinoa protein isolate (QPI) dispersions at different pH values for (**A**) Q1, (**B**) Q2, and (**C**) Q3 after ultracentrifugation. Molecular markers (kDa) were used in the first lane. Corresponding densitometry analyses obtained using ImageJ software are shown in (**D**–**F**), with values presented as mean ± SD (n = 3). Superscript letters indicate a significant (*p* < 0.05) difference (Tukey’s HSD test).

The SDS-PAGE profiles of the QPI supernatant fractions are shown in Figure 4A–C. At pH 3, only bands corresponding to 2S albumin were detected, whereas globulin bands were absent or below the detection limit. Similarly, at pH 5 only faint albumin bands were observed, which were barely visible by visual inspection but could be detected through densitometric analysis. These findings indicate that most quinoa proteins precipitated under acidic conditions, particularly near pH 5, which is close to the isoelectric point of quinoa proteins. Under these conditions, the reduction in net surface charge minimizes electrostatic repulsion, promoting protein–protein interactions and the formation of large, insoluble aggregates.

Conversely, supernatants of QPIs obtained at pH 7, 9, and 11 exhibited the complete profile of quinoa protein subunits, including 2S albumin, 7S globulin, and 11S (chenopodin), represented by their acidic and basic polypeptide chains. The presence of these protein fractions in the soluble phase indicates a substantially greater degree of protein dispersion and solubilization under neutral and alkaline conditions. Furthermore, these observations are consistent with the particle size results (Figure 1), which showed smaller particle sizes at higher pH values, suggesting dissociation of protein aggregates.

Densitometric analysis of the SDS-PAGE gels (4D-F) confirmed a significant (*p* < 0.05) increase in the intensity of soluble protein bands present in the supernatants of QPI aqueous dispersions as pH increased from 7 to 11. This trend was consistent across all QPI samples and demonstrated that alkaline conditions increased the abundance of soluble albumin and globulin fractions. The increased abundance of soluble proteins at elevated pH corresponded with the marked reduction in D_10_, D_50_, and D_90_ values and the increase in surface hydrophobicity observed in Figure 1 and Figure 2A, respectively. Together, the particle size and SDS-PAGE results indicate that alkaline conditions favored protein dispersion, as evidenced by smaller particle sizes and a greater abundance of albumin and globulin polypeptides in the supernatant. Notably, the increased intensity of bands corresponding to 11S globulin (chenopodin) at pH 9 and 11 indicates a greater abundance of this protein fraction in the soluble phase under alkaline conditions. Because SDS-PAGE was performed under reducing and denaturing conditions, and equal sample volumes were loaded, the observed polypeptide profiles primarily reflect differences in the relative abundance of proteins present in the soluble fraction and do not provide direct evidence of the native oligomeric state or dissociation of quinoa protein subunits. This enhanced solubilization of chenopodin, together with the albumin and 7S globulin fractions, may contribute to the improved functional properties often reported for quinoa proteins under alkaline conditions.

### 3.6. Influence of pH on the Soluble-Solid Recovery of QPI Aqueous Dispersions

**Figure 5:**
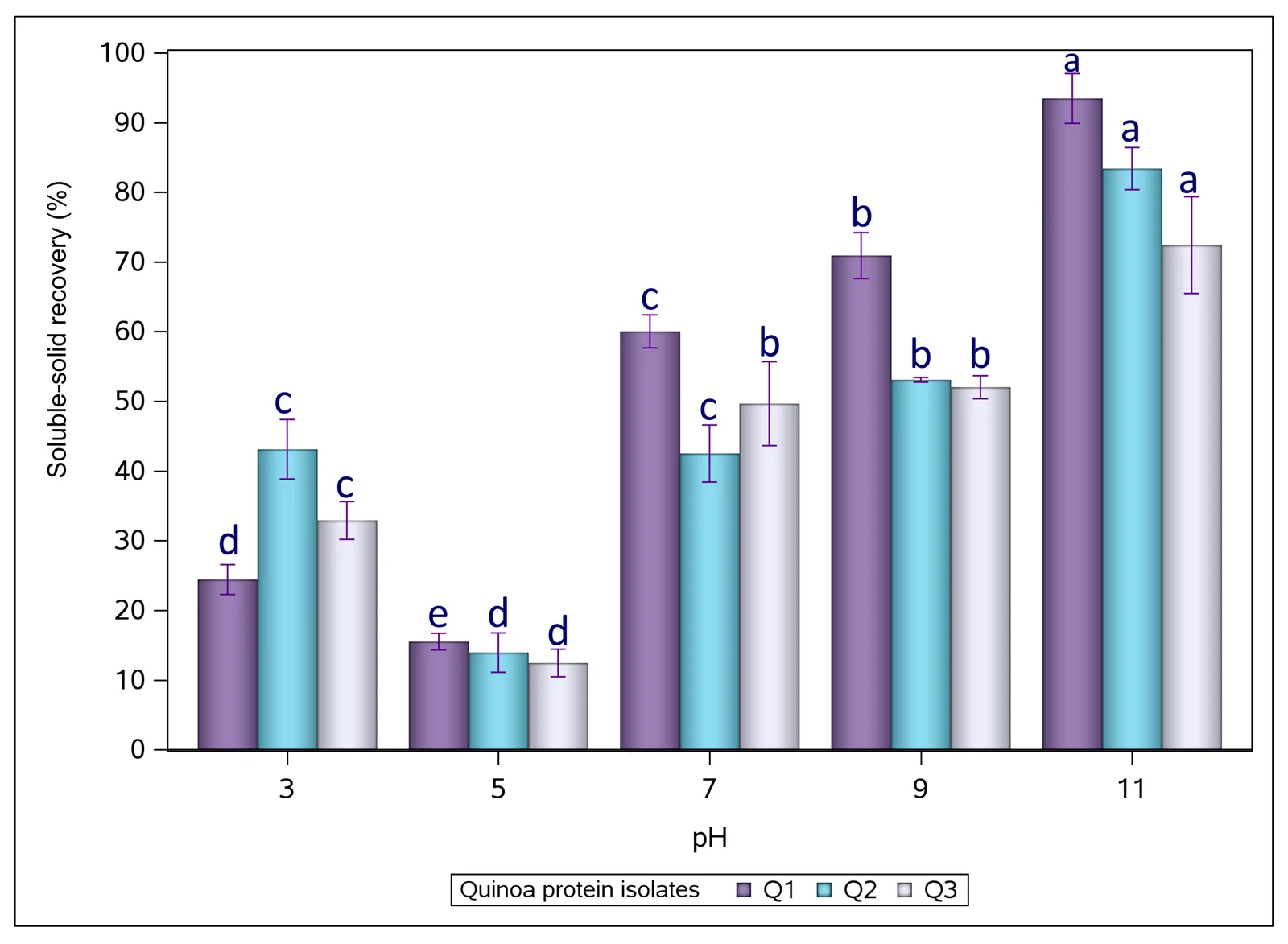
Soluble-solid recovery of QPI aqueous dispersions at different pH conditions. Values and error bars indicate mean ± SD (n = 3). Superscript letters indicate a significant *(p* < 0.05) difference (Tukey’s HSD test).

Soluble-solid recovery provides an indication of the fraction of total solids remaining in the supernatant following ultracentrifugation. As shown in Figure 5, the soluble-solid recovery of QPI aqueous dispersions increased significantly (*p* < 0.05) as pH increased from neutral to alkaline conditions. At neutral pH, the soluble-solid recovery of QPI aqueous dispersions was approximately 60 ± 2%, 43 ± 4%, and 50 ± 6% for Q1, Q2, and Q3, respectively. Soluble-solid recovery increased gradually with increasing pH and as the aqueous dispersion became more alkaline, reaching maximum values at pH 11 of 94 ± 4%, 83 ± 3%, and 72 ± 7% for Q1, Q2, and Q3, respectively. As expected, the lowest soluble-solid recovery values were observed at pH 5 (close to the isoelectric point of quinoa proteins, approximately pH 4.5), where Q1, Q2, and Q3 exhibited soluble-solid recoveries of 16 ± 1%, 14 ± 3%, and 12 ± 2%, respectively. This pH corresponds closely to the isoelectric region of quinoa proteins, where protein–protein interactions are favored, and solubility is minimized. Similar trends have been reported previously for quinoa protein isolates [7,12].

Because the QPI samples were predominantly composed of protein, as determined by the Dumas combustion method (Table 1), the observed changes in soluble-solid recovery are expected to be largely associated with changes in protein dispersion. However, soluble-solid recovery is not protein-specific, and contributions from other soluble constituents, including carbohydrates and minerals, cannot be excluded. Consistent with this trend, SDS-PAGE and densitometric analysis of the supernatant fractions showed an increased abundance of soluble albumin and globulin fractions under neutral and alkaline conditions.

The soluble-solid recovery trends were consistent with the changes in particle size described in Figure 1. Under acidic conditions, reduced electrostatic repulsion promoted the formation of large protein aggregates, resulting in lower protein dispersion and solubility. Conversely, increasing the pH enhanced the ionization of acidic amino acid residues, increasing the net negative charge of the proteins and promoting electrostatic repulsion. This likely facilitated the dissociation of protein aggregates, leading to the smaller particle sizes observed at pH 9 and 11 and contributing to the marked increase in soluble-solid recovery and protein dispersion.

SDS-PAGE analysis of the supernatants of ultracentrifuged QPI dispersions (Figure 4) provided further evidence of the pH-dependent changes in protein solubility. At acidic pH values (3 and 5), only small amounts of soluble proteins were detected, with the soluble fraction consisting primarily of 2S albumin subunits, while globulins were largely absent, indicating extensive precipitation of higher molecular weight fractions. This coincided with the lowest soluble-solid recovery values. In contrast, at pH 7, 9, and 11 the supernatants exhibited a more complete protein profile, including 2S albumin, 7S globulin, and chenopodin (11S), indicating improved protein dispersion and solubility under neutral and alkaline conditions. Densitometric analysis confirmed a significant (*p* < 0.05) increase in the abundance of soluble proteins with increasing pH, particularly under extreme alkaline conditions.

The greater abundance of soluble albumin and globulin fractions, including chenopodin, indicates that these protein fractions were increasingly present in the supernatant under alkaline conditions. This trend was consistent with the smaller particle sizes observed at pH 9 and 11, suggesting greater protein dispersion under alkaline conditions. Increased ionization of acidic amino acid residues and the resulting electrostatic repulsion may contribute to reduced intermolecular association and improved protein dispersion. However, because SDS-PAGE was conducted under reducing and denaturing conditions, the observed polypeptide profiles cannot be used to determine the native oligomeric state of the proteins or to establish dissociation of native protein subunits. Therefore, the SDS-PAGE results are interpreted primarily as evidence of differences in the relative abundance of albumin- and globulin-associated polypeptides in the soluble fraction under the different pH conditions.

### 3.7. Influence of pH on the Emulsifying and Foaming Properties of QPI Aqueous Dispersions

**Figure 6:**
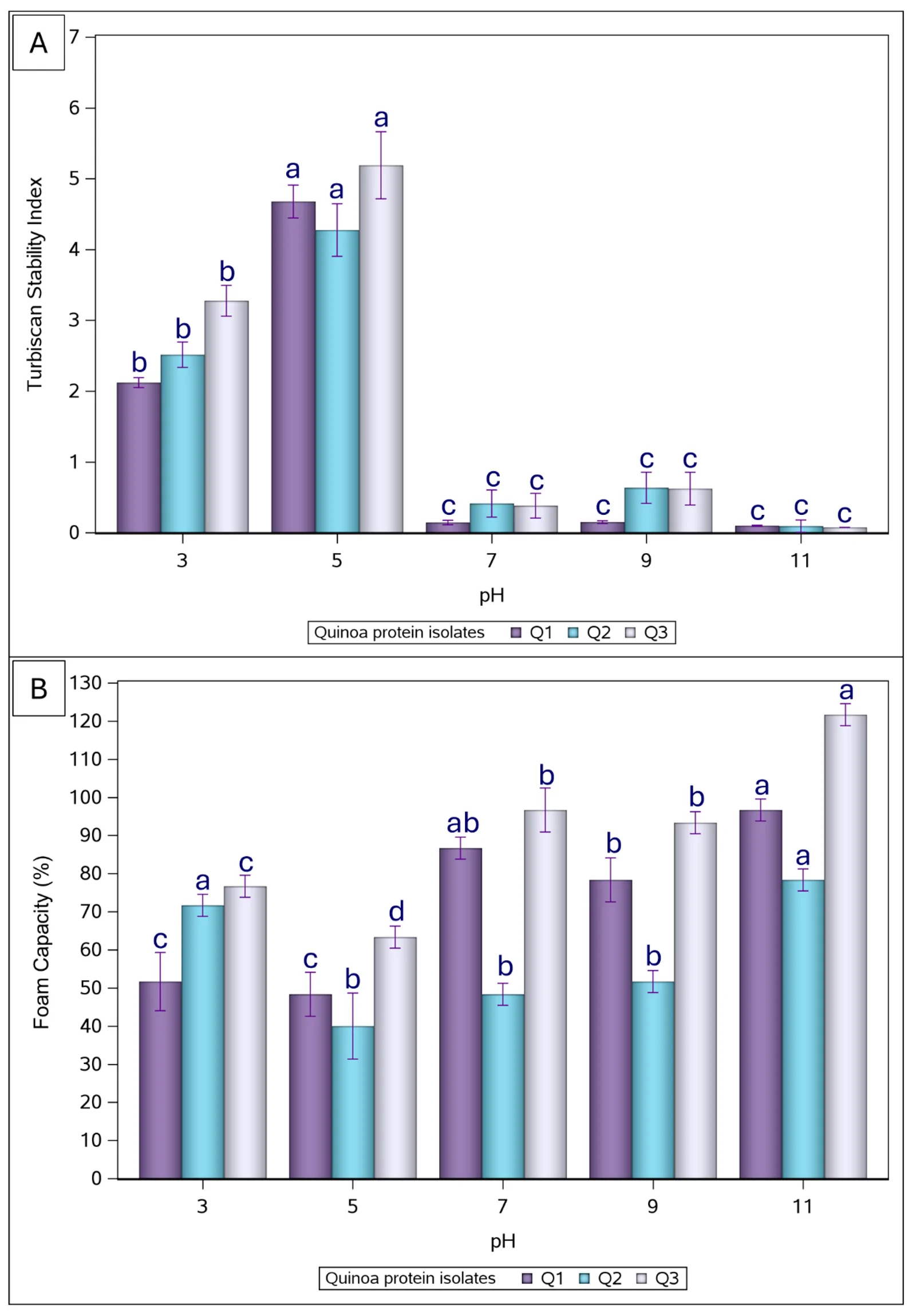
(**A**) Turbiscan Stability Index (TSI) and (**B**) foam capacity of QPI aqueous dispersions prepared at different pH conditions. A lower TSI value indicates greater physical stability of the emulsion. Values and error bars indicate mean ± SD (n = 3). Superscript letters indicate a significant (*p* < 0.05) difference (Tukey’s HSD test).

The Turbiscan Stability Index (TSI) of QPI-stabilized emulsions decreased as pH increased from acidic (pH 3 and 5) to neutral (pH 7) and alkaline (pH 9 and 11) conditions, indicating improved emulsion stability at higher pH values (Figure 6A). No significant differences (*p* > 0.05) were observed among emulsions prepared at pH 7, 9, and 11. Conversely, emulsions prepared under acidic conditions (pH 3 and 5) exhibited significantly higher TSI values (*p* < 0.05), reflecting a greater tendency toward destabilization. The improved stability observed at neutral and alkaline pH may be related to the increased abundance of soluble protein fractions and improved molecular dispersion of quinoa proteins, which could favor protein adsorption at the oil–water interface and the formation of a more stable interfacial protein layer around oil droplets, thereby reducing physical destabilization. It should be noted that initial emulsion droplet-size distribution and ζ-potential were not measured in the present study. Therefore, although TSI provides an overall assessment of physical destabilization, the relative contributions of specific destabilization mechanisms, such as creaming, flocculation, and coalescence, cannot be distinguished. Future studies incorporating droplet-size and ζ-potential measurements would provide further insight into the mechanisms underlying the observed differences in emulsion stability.

The foaming behavior of QPI dispersions, evaluated through foam capacity (FC), followed a similar pH-dependent trend (Figure 6B). At pH 11, QPI dispersions exhibited the highest foaming capacity, with FC values of 97 ± 3%, 79 ± 3%, and 122 ± 3% for Q1, Q2, and Q3, respectively. In contrast, the lowest FC values were observed at pH 5, where extensive aggregation and the low abundance of soluble protein fractions possibly restricted protein diffusion and adsorption at the air–liquid interface. The improved foaming capacity under alkaline conditions may be associated with the smaller and more highly dispersed protein structures observed at high pH, which could facilitate protein diffusion and adsorption at the air–water interface. The increased abundance of soluble albumin and globulin fractions, particularly chenopodin (Figure 4), may have further contributed to foam formation. Greater protein flexibility and rapid adsorption at the air–water interface have similarly been associated with enhanced foam formation in pea protein and lupin protein–polysaccharide systems [35,36].

**Figure 7:**
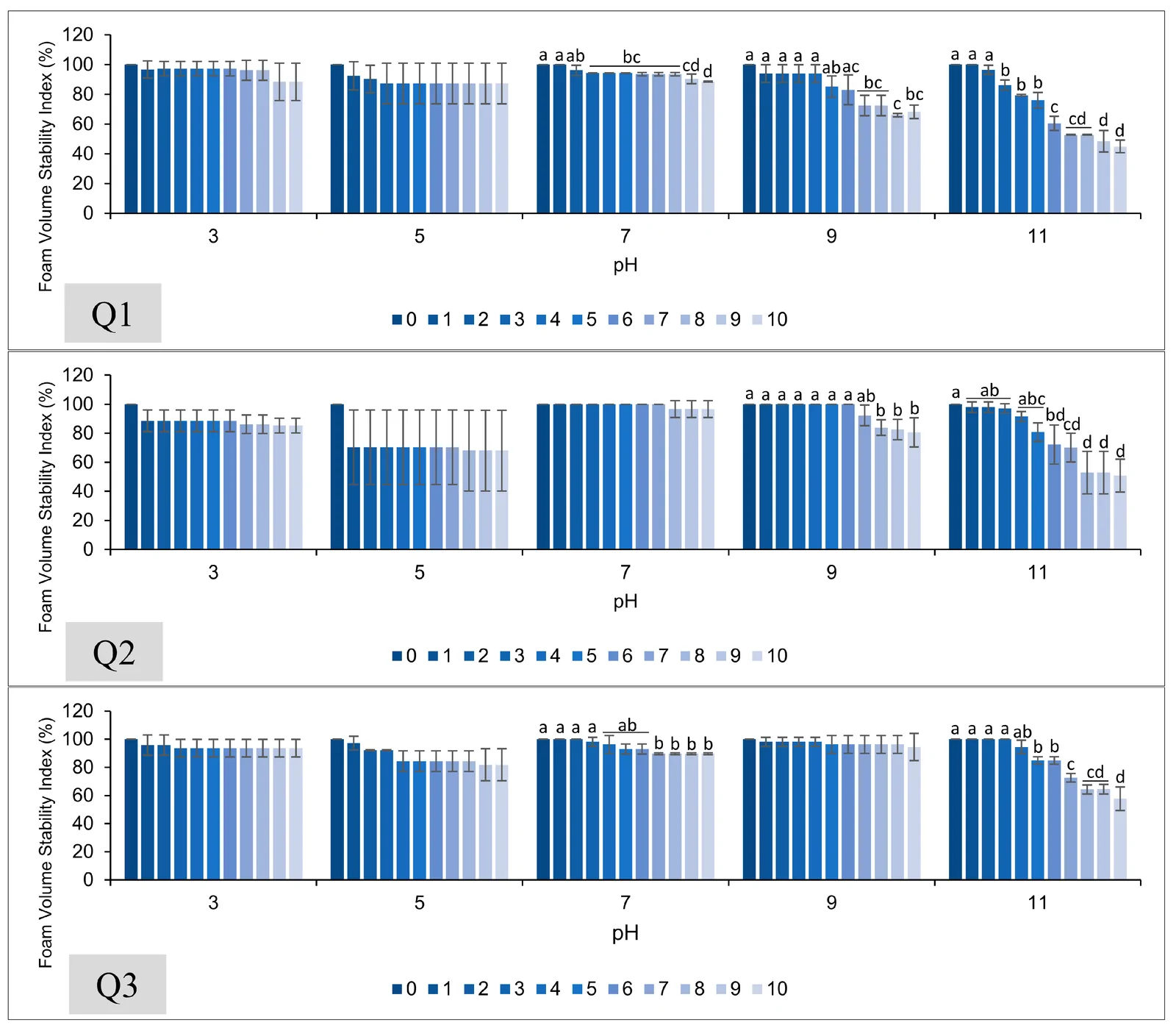
Foam volume stability index (FVSI) values for QPI aqueous dispersions at different pH values over 10 h at 21 °C. Values and error bars indicate mean ± SD (n = 3). Superscript letters indicate a significant (*p* < 0.05) difference (Tukey’s HSD test).

Foam stability, evaluated through the foam volume stability index (FVSI), is a critical parameter for applications requiring prolonged retention of aerated structures. As shown in Figure 7, FVSI exhibited pH-dependent behavior over a 10 h storage period. Foams prepared under acidic and neutral conditions generally exhibited greater retention of their initial foam volume. In contrast, although foams prepared at pH 11 exhibited high initial foaming capacity, their FSVI decreased progressively during the 10 h storage period. However, FVSI values after 10 h remained approximately 45%, 51%, and 58% for Q1, Q2, and Q3, respectively, indicating substantial foam destabilization rather than complete collapse.

These results suggest that the factors promoting foam formation under alkaline conditions were not necessarily conducive to long-term foam stability. Although greater protein flexibility and rapid interfacial adsorption can favor foam formation, prolonged stability also depends on the formation of sufficiently cohesive and viscoelastic interfacial films capable of resisting drainage and bubble coalescence. These relationships have been observed in pea protein, lupin protein–polysaccharide, and amaranth protein systems [35,36,37]. At pH 11, increased electrostatic repulsion may have favored protein dispersion while limiting protein–protein interactions and interfacial cohesion. Supporting this general interfacial mechanism, increased electrostatic repulsion in foams stabilized by rapeseed napin, a plant seed-storage protein, resulted in less densely packed, more fluid interfacial layers and greater susceptibility to coalescence [38]. Thus, the high FC but lower FVSI at pH 11 may reflect a balance between favorable protein dispersion for initial foam formation and insufficient interfacial cohesion for prolonged stabilization.

### 3.8. Principal Component Analysis and Pearson Correlation

**Figure 8:**
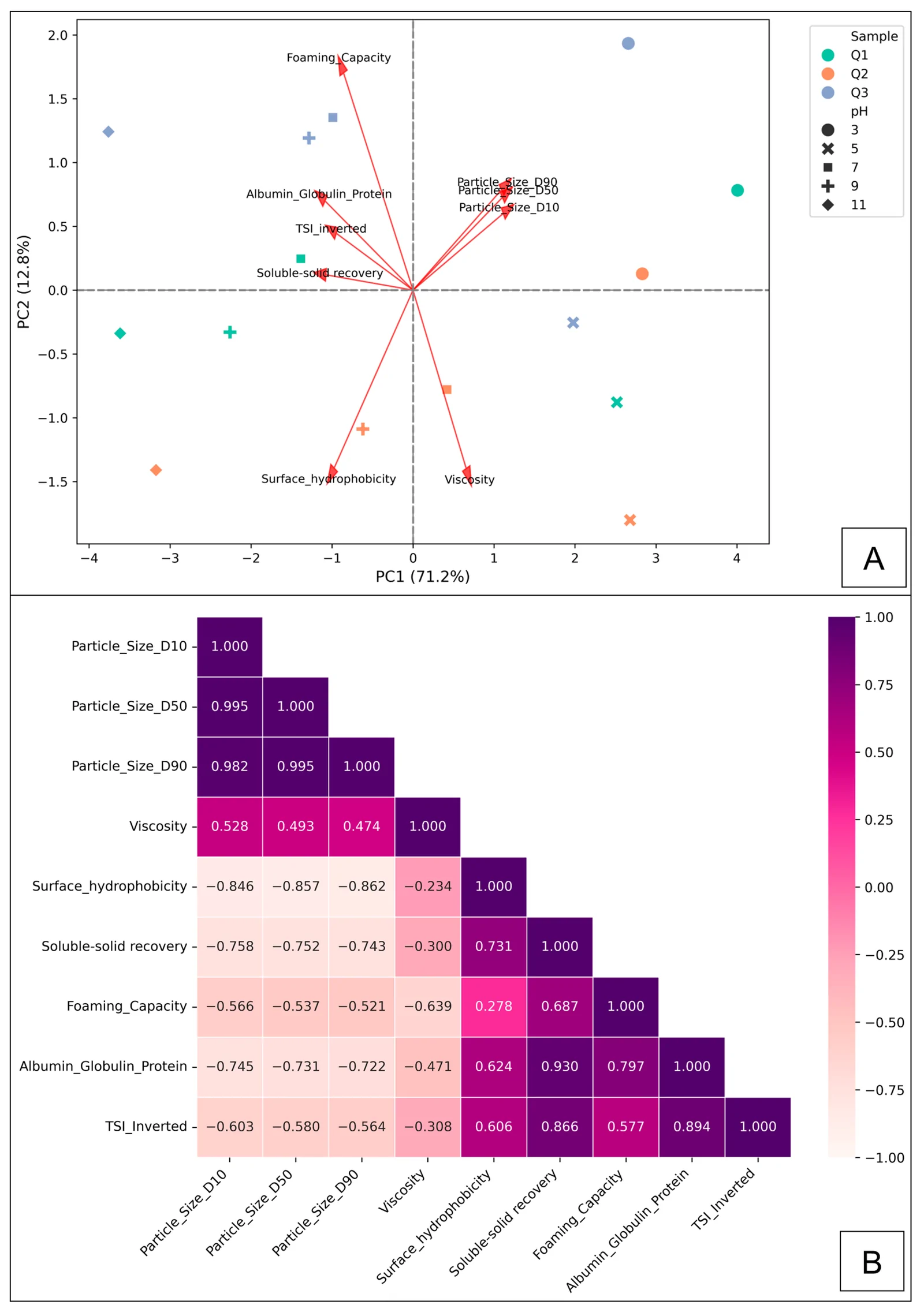
(**A**) Principal Component Analysis (PCA) biplot of QPIs’ physicochemical, structural, and interfacial properties under varying pH conditions (3, 5, 7, 9, 11) and for different samples (Q1, Q2, Q3). Arrows indicate the loading vectors of each variable, showing their contribution and correlation to principal components PC1 (71.2%) and PC2 (12.8%). (**B**) Pearson correlation heatmap of QPIs. Color intensity represents the strength and direction of correlation (from −1 to +1), with darker shades indicating stronger correlations. The Turbiscan Stability Index (TSI) was inverted (TSI_Inverted) to reflect that lower original TSI values correspond to greater emulsion stability.

Figure 8A presents the Principal Component Analysis (PCA) biplot summarizing the relationships among the physicochemical, structural, and interfacial properties of the three quinoa protein isolates (Q1, Q2, Q3) across the pH range studied (3 to 11). The first two principal components (PC1 and PC2) explained 84.0% of the total variance, with PC1 and PC2 accounting for 71.2% and 12.8% of the total variance, respectively. PC1 primarily captured variations related to particle size (D_10_, D_50_, D_90_), viscosity, and surface hydrophobicity, whereas PC2 captured trends associated with soluble-solid recovery, foaming capacity, emulsion stability, and albumin–globulin protein fraction.

A clear separation of samples according to pH was observed. For all three isolates (Q1, Q2, and Q3), dispersions prepared under neutral and alkaline conditions (pH 7, 9, and 11) clustered in regions associated with higher soluble-solid recovery and superior interfacial properties, including enhanced foaming and emulsion stability and a higher abundance of soluble albumin and globulin fractions. In contrast, dispersions under acidic conditions (pH 3 and 5) occupied regions of the biplot associated with protein aggregation, characterized primarily by larger particle sizes and lower soluble-solid recovery. These samples were clearly separated from those exhibiting superior interfacial and functional properties. These multivariate patterns reinforce earlier findings, emphasizing the pivotal role of pH-dependent physicochemical changes in governing protein behavior in aqueous systems. Collectively, the PCA results indicate that protein aggregation, soluble-solid recovery, and the distribution of albumin–globulin fractions are important variables associated with the functional performance of QPI dispersions.

The corresponding Pearson correlation heatmap (Figure 8B) further reinforces these trends, revealing strong negative correlations between particle size and both soluble-solid recovery (r < −0.74) and TSI-inverted values, which reflects emulsion stability (r < −0.60), indicating that the formation of larger protein aggregates reduces emulsion stability and soluble-solid recovery. TSI-inverted value showed a strong positive correlation with soluble-solid recovery (r = 0.87), as well as with the albumin–globulin protein fraction (r = 0.89), highlighting the contribution of soluble proteins to emulsion stabilization.

Notably, the albumin–globulin fraction also exhibited a strong positive correlation with the soluble-solid recovery (r = 0.93) and foaming capacity (r = 0.80), suggesting that these protein subunits are associated with enhanced interfacial functionality by improving protein dispersibility and adsorption at the air–water interface. Collectively, these relationships highlight the central role of soluble protein fractions in promoting both emulsifying and foaming properties, which are essential attributes for the development of plant-based functional ingredients.

Collectively, these integrative analyses demonstrate associations among soluble-solid recovery, particle size, soluble albumin–globulin protein fractions, and functional performance across the pH conditions studied. The consistent relationships observed across the three QPIs provide an integrated comparative assessment of how dispersion characteristics and soluble protein fraction profiles are associated with the pH-dependent interfacial functionality of QPI aqueous dispersions. These relationships further highlight the potential of pH adjustment for modulating quinoa protein functionality in food applications.

### 3.9. Limitations and Future Directions

A limitation of the present study is the use of a relatively low QPI concentration (1% *w*/*v*), which facilitated the investigation of pH-dependent physicochemical and functional changes but may not fully reflect the behavior of quinoa proteins in commercial food formulations, where higher protein concentrations are typically used. Therefore, future studies should evaluate the impact of pH adjustment under conditions that more closely resemble industrial applications, including concentrated protein systems and complex food matrices.

Moreover, future research should also explore the synergistic interactions of QPIs with other food components, such as polysaccharides, polyphenols, and lipids, to better understand their behavior in multiphase and composite systems. Recent studies have shown that complexing QPI hydrolysates with polyphenols like epigallocatechin gallate (EGCG) can enhance emulsion stability and reduce lipid oxidation under varying pH conditions [39]. Similarly, electrostatic complexes formed between QPIs and gelling-type hydrocolloids (e.g., κ-carrageenan, xanthan gum, pectin) can improve emulsion stability, oxidative stability and rheological properties, including enhanced viscoelasticity and printability for advanced food structuring [40].

The results from the present study highlight the versatility of quinoa protein as a sustainable and functional ingredient platform for next-generation food design. Given quinoa’s adaptability to harsh and arid environments, advancing its utilization as a sustainable protein source may contribute to food innovation in regions where traditional crops are less viable.

## 4. Conclusions

Adjusting the pH of quinoa protein isolate (QPI) aqueous dispersions from acidic to neutral and alkaline conditions significantly improved soluble-solid recovery, foaming capacity, and emulsion stability. These improvements were associated with the dissociation of large protein aggregates into smaller dispersed particles and with an increased abundance of soluble albumins and globulin fractions, including chenopodin, resulting in enhanced interfacial activity. SDS-PAGE, correlation, and multivariate analyses supported the conclusion that alkaline conditions promoted the solubilization and dispersion of albumin and globulin protein fractions, particularly chenopodin, thereby contributing to improved functionality. By comparatively evaluating three QPIs under identical pH conditions, this study further demonstrates relationships among their pH-dependent physicochemical and functional responses while revealing differences in pH responsiveness among the isolates. Collectively, these findings indicate that pH adjustment is a promising strategy for modulating the physicochemical and functional behavior of QPI aqueous dispersions and highlight its potential for the development of clean-label food ingredients. Future work should evaluate QPI functionality at higher protein concentrations and investigate interactions with other food components, such as polysaccharides and lipids, to better understand their performance in complex food systems.

## Figures and Tables

**Table 1:** Physical characteristics of freeze-dried quinoa protein isolates.

Freeze-Dried QPIs	Moisture Content (%)	Protein Content (%)
Q1	2.7 ± 0.4 ^b^	85.9 ± 1.3 ^b^
Q2	6.5 ± 0.2 ^a^	89.3 ± 1.2 ^b^
Q3	3.6 ± 0.4 ^b^	96.0 ± 2.3 ^a^

Values are given as mean ± SD (n = 3). Means followed by different superscript letters are significantly different (*p* < 0.05).

## Data Availability

The original contributions presented in the study are included in the article/Supplementary Material, further inquiries can be directed to the corresponding authors.

## Supplementary Materials

The following supporting information can be downloaded at: https://www.mdpi.com/article/10.3390/foods15193501/s1, Figure S1: Density particle size distribution (q3%) plotted on a logarithmic scale for quinoa protein isolates (Q1, Q2, and Q3) aqueous dispersions under different pH conditions.

## Abbreviations

The following abbreviations are used in this manuscript:

D_50_	Median Particle Diameter
FC	Foaming Capacity
FVSI	Foam Volume Stability Index
PCA	Principal Component Analysis
QPI	Quinoa Protein Isolate
SDS-PAGE	Sodium Dodecyl Sulfate-Polyacrylamide Gel Electrophoresis
H_o_	Surface Hydrophobicity
TSI	Turbiscan Stability Index

## References

[1] Aschemann-Witzel J., Gantriis R.F., Fraga P., Perez-Cueto F.J.A. (2020). Plant-Based Food and Protein Trend from a Business Perspective: Markets, Consumers, and the Challenges and Opportunities in the Future. Crit. Rev. Food Sci. Nutr..

[2] Chauhan K., Rao A. (2024). Clean-Label Alternatives for Food Preservation: An Emerging Trend. Heliyon.

[3] Qin P., Wang T., Luo Y. (2022). A Review on Plant-Based Proteins from Soybean: Health Benefits and Soy Product Development. J. Agric. Food Res..

[4] Mintel Plant-Based Proteins: INCL Impact of COVID-19-US-May 2020. https://clients-mintel-com.proxy.lib.ohio-state.edu/?freetext=PLANT-BASEDPROTEINS%3AINCLIMPACTOFCOVID-19-US-MAY2020.

[5] Escuredo O., González Martín M.I., Wells Moncada G., Fischer S., Hernández Hierro J.M. (2014). Amino Acid Profile of the Quinoa (*Chenopodium quinoa* Willd.) Using near Infrared Spectroscopy and Chemometric Techniques. J. Cereal Sci..

[6] Mir N.A., Riar C.S., Singh S. (2019). Effect of pH and Holding Time on the Characteristics of Protein Isolates from *Chenopodium* Seeds and Study of Their Amino Acid Profile and Scoring. Food Chem..

[7] Dakhili S., Abdolalizadeh L., Hosseini S.M., Shojaee-Aliabadi S., Mirmoghtadaie L. (2019). Quinoa Protein: Composition, Structure and Functional Properties. Food Chem..

[8] Opazo-Navarrete M., Schutyser M.A.I., Boom R.M., Janssen A.E.M. (2018). Effect of Pre-Treatment on in Vitro Gastric Digestion of Quinoa Protein (*Chenopodium quinoa* Willd.) Obtained by Wet and Dry Fractionation. Int. J. Food Sci. Nutr..

[9] Turkut G.M., Cakmak H., Kumcuoglu S., Tavman S. (2016). Effect of Quinoa Flour on Gluten-Free Bread Batter Rheology and Bread Quality. J. Cereal Sci..

[10] Yildiz G., Yıldız G. (2023). A New Approach to Enhance Quinoa Protein Nano-Aggregates: Combined pH Shifting—High Pressure Homogenization. Food Chem..

[11] Mu H., Xue S., Sun Q., Shi J., Zhang D., Wang D., Wei J. (2023). Research Progress of Quinoa Seeds (*Chenopodium quinoa* Wild.): Nutritional Components, Technological Treatment, and Application. Foods.

[12] Ruiz G.A., Xiao W., Van Boekel M., Minor M., Stieger M. (2016). Effect of Extraction pH on Heat-Induced Aggregation, Gelation and Microstructure of Protein Isolate from Quinoa (*Chenopodium quinoa* Willd). Food Chem..

[13] Kaspchak E., de Oliveira M.A.S., Simas F.F., Franco C.R.C., Silveira J.L.M., Mafra M.R., Igarashi-Mafra L. (2017). Determination of Heat-Set Gelation Capacity of a Quinoa Protein Isolate (*Chenopodium quinoa*) by Dynamic Oscillatory Rheological Analysis. Food Chem..

[14] Van de Vondel J., Janssen F., Wouters A.G.B., Delcour J.A. (2023). Air-Water Interfacial and Foaming Properties of Native Protein in Aqueous Quinoa (*Chenopodium quinoa* Willd.) Extracts: Impact of pH- and Heat-Induced Aggregation. Food Hydrocoll..

[15] Tang Y.R., Stone A.K., Wang Y., Zhou L., Kimmel J., House J.D., Nickerson M.T. (2023). Effect of a pH Shift Treatment on the Functional Properties of Individual and Blended Commercial Plant Protein Ingredients. Eur. Food Res. Technol..

[16] Elsohaimy S.A., Refaay T.M., Zaytoun M.A.M. (2015). Physicochemical and Functional Properties of Quinoa Protein Isolate. Ann. Agric. Sci..

[17] Cui H., Roy D., Li S., Loo T.S., Guo Q., Ye A. (2025). Recovery and Characterisation of Quinoa Protein via Neutral to Alkaline Extraction: Protein Profiles and Heat-Induced Aggregation Behaviour. Curr. Res. Food Sci..

[18] Wang H., Cheng L., Yang H., Shen Q., Yang Z. (2025). Effect of pH-Shifting Combined with Heat or Ultrasonication Treatment on Physicochemical Properties of Quinoa Protein Isolates (QPI) Dispersions. Int. J. Biol. Macromol..

[19] Solanilla-Duque F., Roa-Acosta D.F., Bravo J.E. (2025). Effect of pH and Concentration on Physicochemical, Adsorption Kinetics and Rheology Properties of Quinoa Protein: Functional Correlations. JCIS Open.

[20] Föste M., Elgeti D., Brunner A.K., Jekle M., Becker T. (2015). Isolation of Quinoa Protein by Milling Fractionation and Solvent Extraction. Food Bioprod. Process..

[21] Abugoch L.E., Romero N., Tapia C.A., Silva J., Rivera M. (2008). Study of Some Physicochemical and Functional Properties of Quinoa (*Chenopodium quinoa* Willd) Protein Isolates. J. Agric. Food Chem..

[22] Shen Y., Tang X., Li Y. (2021). Drying Methods Affect Physicochemical and Functional Properties of Quinoa Protein Isolate. Food Chem..

[23] Astorga-Oquendo L. (2021). Effect of High-Pressure Jet Processing on the Structure and Physicochemical Properties of Plant Protein Isolates in Aqueous Dispersions. Master’s Thesis.

[24] Jung S., Rickert D.A., Deak N.A., Aldin E.D., Recknor J., Johnson L.A., Murphy P.A. (2003). Comparison of Kjeldahl and Dumas Methods for Determining Protein Contents of Soybean Products. JAOCS J. Am. Oil Chem. Soc..

[25] Haskard C.A., Li-Chan E.C.Y. (1998). Hydrophobicity of Bovine Serum Albumin and Ovalbumin Determined Using Uncharged (PRODAN) and Anionic (ANS-) Fluorescent Probes. J. Agric. Food Chem..

[26] Astorga-Oquendo L., Lewis G., Mahdinia E., Harte F. (2023). Effect of High-Pressure Jet Processing on the Structure and Physicochemical Properties of Plant Protein Isolate Aqueous Dispersions. Food Hydrocoll..

[27] Steffolani M.E., Villacorta P., Morales-Soriano E.R., Repo-Carrasco R., León A.E., Pérez G.T. (2016). Physicochemical and Functional Characterization of Protein Isolated from Different Quinoa Varieties (*Chenopodium quinoa* Willd.). Cereal Chem..

[28] Vilcacundo R., Barrio D., Carpio C., García-Ruiz A., Rúales J., Hernández-Ledesma B., Carrillo W. (2017). Digestibility of Quinoa (*Chenopodium quinoa* Willd.) Protein Concentrate and Its Potential to Inhibit Lipid Peroxidation in the Zebrafish Larvae Model. Plant Foods Hum. Nutr..

[29] Chen N., Zhao M., Chassenieux C., Nicolai T. (2016). Structure of Self-Assembled Native Soy Globulin in Aqueous Solution as a Function of the Concentration and the pH. Food Hydrocoll..

[30] Hua Y., Cui S.W., Wang Q., Mine Y., Poysa V. (2005). Heat Induced Gelling Properties of Soy Protein Isolates Prepared from Different Defatted Soybean Flours. Food Res. Int..

[31] Zhang W., Liu C., Zhao J., Ma T., He Z., Huang M., Wang Y. (2021). Modification of Structure and Functionalities of Ginkgo Seed Proteins by pH-Shifting Treatment. Food Chem..

[32] Jiang J., Chen J., Xiong Y.L. (2009). Structural and Emulsifying Properties of Soy Protein Isolate Subjected to Acid and Alkaline pH-Shifting Processes. J. Agric. Food Chem..

[33] Dickinson E. (2010). Food Emulsions and Foams: Stabilization by Particles. Curr. Opin. Colloid Interface Sci..

[34] Brinegar C., Goundan S. (1993). Isolation and Characterization of Chenopodin, the 11S Seed Storage Protein of Quinoa (*Chenopodium quinoa*). J. Agric. Food Chem..

[35] Wu J., Zhou Z., Su F., Guo W., Wu X. (2026). Food Hydrocolloids Comparative Analysis of Extraction Techniques Influences Pea Protein Foam Stability via Interfacial Behavior. Food Hydrocoll..

[36] Ma X., Habibi M., Sagis L.M.C. (2025). Food Hydrocolloids Air-Water Interfacial and Foaming Properties of Lupin Protein-Polysaccharide Soluble Complexes: Role of Physicochemical Properties, Morphological Characteristics, and Flexibility. Food Hydrocoll..

[37] Bolontrade A.J., Scilingo A.A., María C.A. (2016). Amaranth Proteins Foaming Properties: Film Rheology and Foam Stability—Part 2. Colloids Surf. B Biointerfaces.

[38] Zondervan S.J.M., Chatzigiannakis E., Mitrofan I., Bitter J.H., Keppler J.K., Nikiforidis C. (2026). Janus-like Napins for Switchable Protein Foam Stability. J. Colloid Interface Sci..

[39] Xu J., Zhang H., Deng M., Guo H., Cui L., Liu Z., Xu J. (2024). Formation Mechanism of Quinoa Protein Hydrolysate-EGCG Complexes at Different pH Conditions and Its Effect on the Protein Hydrolysate-Lipid Co-Oxidation in Emulsions. Food Res. Int..

[40] Zhong W., Wang Q., Shen X. (2024). Quinoa Protein/Polysaccharide Electrostatic Complex Stabilized Vegan High Internal Phase Emulsions for 3D Printing: Role of Complex State and Gelling-Type Polysaccharides. Food Chem..

