# Supplementary material for: pH-Dependent Changes in the Structural and Functional Properties of Quinoa Protein Isolates in Aqueous Dispersions
Source: Foods. 2026 Sep 30;15(19):3501. doi: 10.3390/foods15193501 (PMC13664372; doi:10.3390/foods15193501)
Supplement: Supplementary file 1 [file foods-15-03501-s001.zip › foods-4527467-supplementary.pdf]

Supplementary material

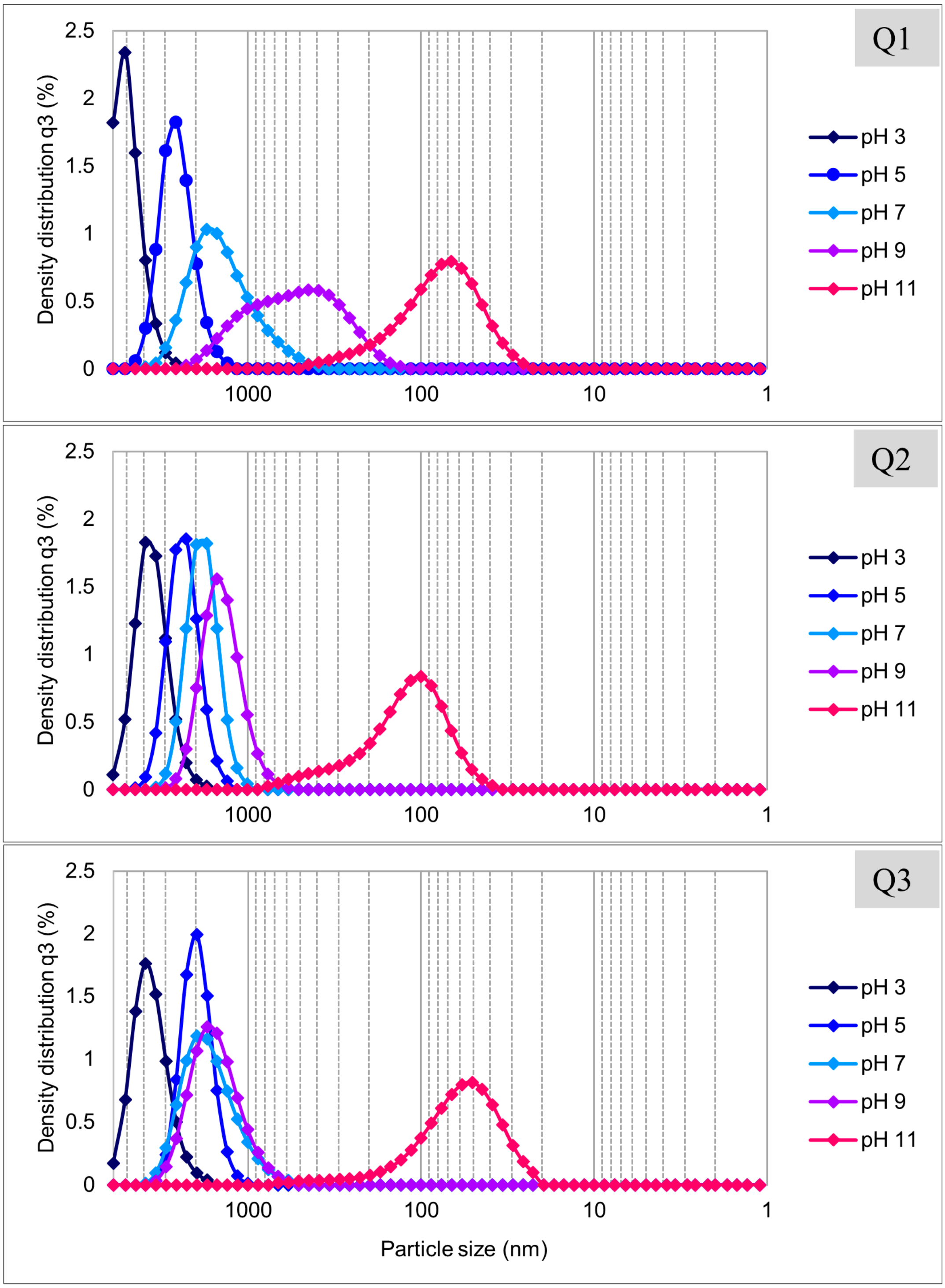

Figure S1. Density particle size distribution (q3%) plotted on a logarithmic scale for quinoa protein isolates (Q1, Q2, and Q3) aqueous dispersions under different pH conditions.
